# *Irx3* is required for postnatal maturation of the mouse ventricular conduction system

**DOI:** 10.1038/srep19197

**Published:** 2016-01-20

**Authors:** Kyoung-Han Kim, Anna Rosen, Samer M. I. Hussein, Vijitha Puviindran, Adam S. Korogyi, Carmelina Chiarello, Andras Nagy, Chi-chung Hui, Peter H. Backx

**Affiliations:** 1 Program in Developmental & Stem Cell Biology, The Hospital for Sick Children, Toronto, ON M5G 0A4, Canada; 2 The Heart and Stroke Richard Lewar Centre of Excellence, University of Toronto, Toronto, ON M5S 3E2, Canada; 3 Departments of Physiology and Medicine, University of Toronto, Toronto, ON M5S 1A8, Canada; 4 Lunenfeld-Tanenbaum Research Institute, Mount Sinai Hospital, Toronto, Ontario M5G 1X5, Canada; 5 Centre Hospitalier Universitaire de Québec Research Center and Faculty of Medicine, Laval University, Quebec City, QC G1V 4G2, Canada; 6 Institute of Medical Science and Department of Obstetrics and Gynaecology, University of Toronto, Toronto, Ontario M5T 3H7, Canada; 7 Department of Molecular Genetics, University of Toronto, Toronto, ON M5S 1A8, Canada; 8 Peter Munk Cardiac Centre and Division of Cardiology, University Health Network, Toronto ON.

## Abstract

The ventricular conduction system (VCS) orchestrates the harmonious contraction in every heartbeat. Defects in the VCS are often associated with life-threatening arrhythmias and also promote adverse remodeling in heart disease. We have previously established that the *Irx3* homeobox gene regulates rapid electrical propagation in the VCS by modulating the transcription of gap junction proteins Cx40 and Cx43. However, it is unknown whether other factors contribute to the conduction defects observed in *Irx3* knockout (*Irx3*^−/−^) mice. In this study, we show that during the early postnatal period, *Irx3*^−/−^ mice develop morphological defects in the VCS which are temporally dissociated from changes in gap junction expression. These morphological defects were accompanied with progressive changes in the cardiac electrocardiogram including right bundle branch block. Hypoplastic VCS was not associated with increased apoptosis of VCS cardiomyocytes but with a lack of recruitment and maturation of ventricular cardiomyocytes into the VCS. Computational analysis followed by functional verification revealed that Irx3 promotes VCS-enriched transcripts targeted by Nkx2.5 and/or Tbx5. Altogether, these results indicate that, in addition to ensuring the appropriate expression of gap junctional channels in the VCS, *Irx3* is necessary for the postnatal maturation of the VCS, possibly via its interactions with Tbx5 and Nkx2.5.

The ventricular conduction system (VCS), comprised of His-bundle, bundle branches, and Purkinje fiber network, plays a critical role in rapid electric propagation through the ventricles of the heart^1^. It ensures proper electrical activation from apex to base, which orchestrates efficient contraction and pumping of the chambers of the heart. Defects in the VCS are clinically important, either in isolation or in association with heart failure, due to promotion of arrhythmias and acceleration of disease processes^2^.

Development of the VCS is coordinated by the appropriate spatiotemporal activation of several cardiac transcription factors, such as Nkx2.5^3^,^4^, Tbx3^5^,^6^, Tbx5^7^, Id2^8^, HF-1b^9^ and Hopx^10^. Loss of these transcription factors leads to various conduction defects including atrioventricular block and bundle branch blocks. Of particular note for our studies is the observation that haploinsufficiency of *Nkx2.5* (*Nkx2.5*^+/−^) or *Tbx5* (*Tbx5*^+/−^) leads to conduction defects with increased susceptibility to arrhythmias^4^,^11^ in association with anatomical hypoplasia of the cardiac conduction system^4^,^7^,^12^. The limited impact of *Nkx2.5* and *Tbx5* deficiency on the VCS, despite the expression of these transcription factors through the working myocardium suggests that some unknown VCS-specific molecules cooperate with Nkx2.5 and Tbx5 in the regulation of VCS function and development.

Recently, we have demonstrated that *Irx3*, a member of the *Iroquois* family of transcription factors^13^, regulates electrical propagation of the ventricles^14^ by modulating the transcription of gap junction genes in the VCS (i.e. *Gja5* and *Gja1* which encode for Cx40 and Cx43, respectively). In addition, a recent study has identified two novel *IRX3* mutations in patients with idiopathic ventricular fibrillation and demonstrated that these mutations resulted in impaired transcriptional regulation of *Gja5*^15^. However, it was not clear whether the right bundle branch block (RBBB) as well as the pattern of reduced ventricular conduction velocity seen in these studies could be explained entirely by abnormal gap junction expression. Specifically, we found that, consistent with previous studies^16^,^17^, the conduction deficiencies seen in Cx40 heterozygous mutant mice were not as severe as seen in mice lacking *Irx3* despite having similar reductions in Cx40 expression. In the present study, we show that *Irx3*^−/−^ mice exhibit progressive changes in electrocardiogram (ECG) recordings during the early postnatal period, which is accompanied by structural deterioration of the VCS, similar to the developmental changes observed in *Nkx2.5*^+/−^ mice^4^,^12^ and that these defects are temporally distinct from the effects of the loss of *Irx3* on gap junction expression. Furthermore, we observed that Irx3 interacts with Tbx5, in addition to Nkx2.5 as we showed previously^14^, and demonstrate that Irx3 regulates the expression of VCS-enriched genes to which Nkx2.5 and/or Tbx5 bind. Together, these results suggest that Irx3 plays an essential role in the postnatal maturation of the VCS, possibly via its interactions with Tbx5 and Nkx2.5.

## Results

### Loss of *Irx3* leads to structural defects in the ventricular conduction system of adult mouse heart

**Figure 1:**
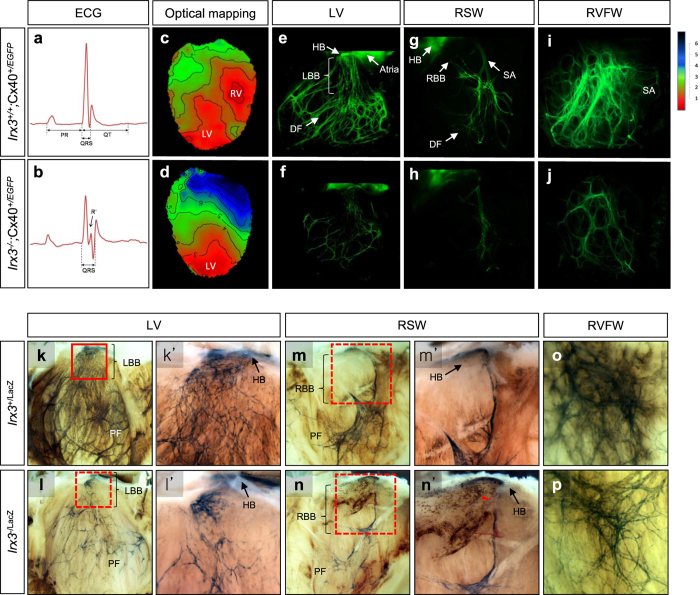
Loss of *Irx3* leads to abnormal electrical activation of the ventricles and morphological defects of the VCS. (**a**,**b**) Representative surface ECG traces show QRS prolongation and notched R’ wave in 10–12 week old *Irx3*^−/−^;Cx40^+/EGFP^ mice (**b**) compared to *Irx3*^+*/*+^;Cx40^+/EGFP^ mice (**a**–**d**). Optical mapping results in the apical four-chamber view. Isochrone lines with 0.5 ms intervals mark areas where depolarization reached 50% intensity. *Irx3*^+*/*+^;Cx40^+/EGFP^ hearts show simultaneous electrical breakthroughs in both LV and RV with depolarization proceeding in an apex-to-base direction, whereas *Irx3*^−/−^;Cx40^+/EGFP^ hearts show a breakthroughs only present in left ventricle (LV) and lack in right ventricle (RV), suggestive of RBB block. (**e**–**j**) Fluorescence images of the VCS visualized using 10–12 week old adult mice expressing Cx40-promoter driven EGFP (Cx40^+/EGFP^). Compared to *Irx3*^+*/*+^;Cx40^+/EGFP^ mice (**e**,**g**,**i**), *Irx3*^−/−^;Cx40^+/EGFP^ mice show reduced fiber densities and fluorescence intensity in the VCS of the left ventricle (**f**) and right ventricular septal and free walls (RSW and RVFW, respectively) (**h,j**). The morphology of the VCS was further compared between *Irx3*^+/LacZ^ and *Irx3*^−/LacZ^ mice. In *Irx3*^+/LacZ^ heart, β-gal staining of *Irx3* expressing cells marked His-Purkinje system in LV (**k**) RSW (**m**) and RVFW (**o**). On the other hand, VCS morphology of *Irx3*^−/LacZ^ heart was severely compromised in LV (**l**) RSW (**n**) and RVFW (**p**) with reduced number and density of fibers in bundle branches and distal Purkinje fiber networks. As marked by a red arrowhead (**n’**), RBB of *Irx3*^−/LacZ^ heart is found disconnected in many cases. **k’**,**l’**,**m’**,**n’** illustrate magnification of framed in panels. HB, His-bundle; LBB, left bundle branch; DF, distal fiber; RBB, right bundle branch; SA, septal artery; and PF, Purkinje fibers.

We previously established that *Irx3*^−/−^ mice showed ~2-fold reductions in Cx40, the major gap junction channel in the VCS, in association with delayed ventricular activation with prolonged QRS intervals and RBBB in 83% of the mice^14^. On the other hand, previous studies^17^,^18^, as well as the data presented below, revealed that mice lacking one Cx40 allele, which are expected to have a ~50% reduction in Cx40 expression, do not show noticeable electrophysiological defects. These findings^18^ suggest that the electrical disturbances seen in *Irx3*^−/−^ mice cannot be explained solely by reductions in Cx40 expression. Given the fact that Irx3 interacts with Nkx2.5^14^ and that conduction defects in mice with *Nkx2.5* haploinsufficiency are linked to developmental deterioration of the His-Purkinje structure^4^,^7^,^8^,^12^, we considered the possibility that *Irx3* might also regulate VCS morphology. To test this hypothesis, we crossed *Irx3*^−/−^ mice with mice expressing GFP under the *Gja5* (Cx40) promoter (i.e. Cx40^+/EGFP^) to allow visualization of the VCS^16^. As shown in previous studies^16^,^17^, adult Cx40^+/EGFP^ mice at 10–12 weeks of age, which have one Cx40 allele along with two wild-type *Irx3* alleles (i.e. *Irx3*^+/+^;Cx40^+/EGFP^), displayed normal surface ECG signals, indistinguishable from wild type (*Irx3*^+/+^;Cx40^+/+^) mice (Fig. 1a and Supplementary Table 1). On the other hand, the ECG recordings in Cx40^+/EGFP^ mice lacking *Irx3* (i.e. *Irx3*^−/−^;Cx40^+/EGFP^) were characterized by prolonged QRS intervals accompanied by R-notches (R’) (Fig. 1b), as expected for mice lacking *Irx3*^14^. In addition, *Irx3*^−/−^;Cx40^+/EGFP^ ventricles had slower ventricular activation patterns as well as right bundle branch block (i.e. no ‘breakthrough’ in the right ventricle; RBBB), which is never observed in *Irx3*^+/+^;Cx40^+/EGFP^ ventricles (Fig. 1c, d).

By imaging the GFP fluorescence images of the left and right bundle branches as well as Purkinje fiber networks, using a previously published technique^16^, we found that *Irx3*^−/−^;Cx40^+/EGFP^ mouse hearts had marked reductions in VCS fiber densities throughout the ventricles compared to those of *Irx3*^+/+^;Cx40^+/EGFP^ hearts (Fig. 1e–j). In addition, the right bundle branch was absent in many *Irx3*^−/−^;Cx40^+/EGFP^ hearts, which correlated with, and explained RBBB in these mice (Fig. 1h). In the remaining VCS fibers, GFP fluorescence intensity was reduced by 58 ± 3% in the left bundle branches and by 35.1 ± 4.5% in the distal fibers of *Irx3*^−/−^;Cx40^+/EGFP^ hearts compared to their counterparts in the *Irx3*^+/+^;Cx40^+/EGFP^ mice (Supplementary Fig. 1). These reductions of fluorescence intensity in the fibers of *Irx3*^−/−^;Cx40^+/EGFP^ hearts are consistent with our previous study showing that *Irx3* positively regulates Cx40 gene expression^14^. Indeed, the fluorescence of Cx40 promoter-dependent GFP expression are not different between *Irx3*^+/+^;Cx40^+/EGFP^ and *Irx3*^−/−^;Cx40^+/EGFP^ mice in either the atria or coronary arteries, which both express Cx40 but not *Irx3*. Furthermore, *Irx3*^+/−^;Cx40^+/EGFP^ mice did not show reductions in the density or fluorescence intensity of fibers in the VCS, compared to *Irx3*^+/+^;Cx40^+/EGFP^ mice, suggesting that the loss of one *Irx3* allele is insufficient to cause a measurable decrease in Cx40 promoter activity.

**Figure 2:**
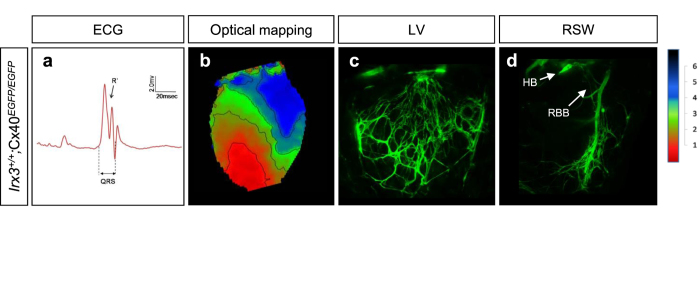
Loss of Cx40 results in abnormal electrical activation of the ventricles without morphological defects of the VCS. **(a**) Representative surface ECG trace shows QRS prolongation and notched R’ wave in *Irx3*^+/+^;Cx40^EGFP/EGFP^ mice. (**b)** Optical mapping in *Irx3*^+/+^;Cx40^EGFP/EGFP^ heart exhibited RBB block, similar to *Irx3*^−/−^ heart. (**c**,**d)** VCS structures visualized by Cx40-EGFP in both LV (**c**) and RSW (**d**) are normal in *Irx3*^+/+^;Cx40^EGFP/EGFP^ mice. LV, left ventricle; RSW, right ventricular septal wall; HB, his-bundle; RBB, right bundle branch.

Since the morphological measurements made using GFP to visualize the VCS could be influenced by the known effects of *Irx3* on the Cx40 promoter^14^, we sought to further confirm a role for *Irx3* in VCS morphology using mice expressing *LacZ* was under the control of the *Irx3* promoter in place of *Irx3*. These mice express one *LacZ* allele and one *Irx3* allele (*Irx3*^+/LacZ^) which allowed robust delineation of the VCS, characterized by an asymmetric morphology of the Bundle of His with numerous fascicles in the left bundle branch (LBB) and a single fascicle in the RBB along with an elaborate Purkinje fiber network (Fig. 1k,m,o). In contrast to the Cx40^+/EGFP^ mice, *Irx3*^+/LacZ^ hearts showed no lacZ staining in either the atria or coronary arteries. More importantly, the *Irx3*^−/LacZ^ mice showed marked reductions in the number of LBB fascicles and Purkinje fibers (Fig. 1l, p), as seen in the *Irx3*^−/−^;Cx40^+/EGFP^ mice. *Irx3*^−/LacZ^ mice also showed disconnections within, or the complete absence of, RBB (Fig. 1n and Supplemental Fig. 2). Together, these results suggest that Irx3 is required for the maintenance of the VCS and that the morphological defects in the VCS seen in *Irx3*^−/−^ mice do not arise from changes in the Cx40 expression levels. This suggestion is further supported by studies in mice lacking Cx40 (i.e. *Irx3*^+/+^;Cx40^EGFP/EGFP^). Since Cx40 is the major connexin in the VCS^16^,^17^, *Irx3*^+/+^;Cx40^EGFP/EGFP^ mice exhibited, as expected, conduction defects, characterized by prolonged QRS durations with R notches (R’) in ECG and abnormal activation patterns with right bundle branch (RBB) block in optical mapping (Fig. 2a,b). Importantly, these cardiac conduction defects in *Irx3*^+/+^;Cx40^EGFP/EGFP^ mice are remarkably similar to those seen in *Irx3*^−/−^ mice, which have only ~2-fold reduction in Cx40. On the other hand, the morphology of the VCS in *Irx3*^+/+^;Cx40^EGFP/EGFP^ mice was indistinguishable from wild-type mice with well-formed bundle branches and distal fibers (Fig. 2c,d). These data support the conclusion that the conduction defects seen in *Irx3*^−/−^ mice are not solely related to reduced Cx40 expression but arise, at least in part, from simultaneous structural changes in the VCS.

### *Irx3* is required for normal function of the VCS during postnatal development

*Irx* genes play key regulatory roles during embryonic development, including patterning, specification, and differentiation of various tissues and organs^13^,^18^,^19^. To determine the time course of the VCS morphological defects in *Irx3*^−/−^ hearts, we first examined mutant hearts at embryonic day 15.5 (E15.5) and found no differences between *Irx3*^−/−^ and *Irx3*^+/+^ control hearts (Supplementary Fig. 3). However, as seen both in postnatal day 5 (P5) and in adult (10–12 week old) *Irx3*^−/−^ hearts^14^, Cx43 expression was ectopically expressed in the proximal bundle branches in *Irx3*^−/−^ hearts at E15.5, which is a developmental stage when Cx43 is normally expressed in myocardium but not in the VCS bundle branches^5^,^20^ (Supplementary Fig. 4). These results are consistent with the conclusion that the regulation of gap junction channel expression in the VCS by Irx3 begins relatively early in embryonic development and continues into adulthood.

**Figure 3:**
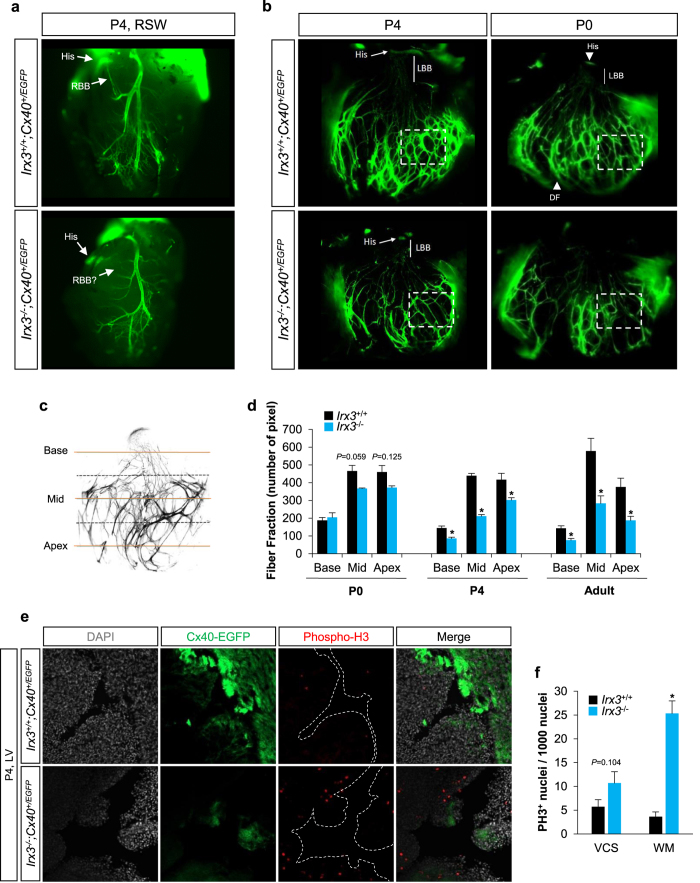
*Irx3* is required for postnatal formation of the VCS. (**a**) Representative images of right bundle branch (RBB) in *Irx3*^+/+^;Cx40^+/EGFP^ and *Irx3*^−/−^;Cx40^+/EGFP^ mice at postnatal day 4 (P4). Note that RBB is absent in many cases when *Irx3* is lost. (**b)** Representative images of the VCS in left ventricle of *Irx3*^+/+^;Cx40^+/EGFP^ and *Irx3*^−/−^;Cx40^+/EGFP^ mice at P4 and P0. *Irx3*^−/−^;Cx40^+/EGFP^ mouse heart exhibit progressive deterioration of the VCS, characterized by reduced fiber density and thickness, compared to P0 heart which show only a mild structural defect. (**c)** A schematic diagram illustrates three different levels of the VCS (i.e. base, mid and apex) for analyzing VCS morphology by counting the number of fiber fraction. (**d)** Quantification of fiber fraction at three levels of the VCS (i.e. base, mid and apex) in three different developmental stages (i.e. P0, P4 and adult). (P0, *Irx3*^+/+^/*Irx3*^−/−^, n = 5/3; P4, *Irx3*^+/+^/*Irx3*^−/−^, n = 3/4; and adult, *Irx3*^+/+^/*Irx3*^−/−^, n = 6/4) *P < 0.05 vs. *Irx3*^+/+^;Cx40^+/EGFP^. (**e**) Representative phospho-histone H3 (PH3) immunofluorescence in the left ventricle of P4 *Irx3* mutant mouse heart expressing Cx40-EGFP that marks the VCS cells. (**f**) Quantification of PH3-positive cells revealed significantly higher number of proliferating cells in *Irx3*^−/−^;Cx40^+/EGFP^ hearts, mainly in EGFP-negative myocardium, compared to *Irx3*^+/+^;Cx40^+/EGFP^ hearts. n = 4. *P < 0.05 vs. *Irx3*^+/+^;Cx40^+/EGFP^ hearts.

Given the fact that Nkx2.5 regulates early postnatal maturation of VCS^12^ and that Nkx2.5 physically interacts with Irx3^14^, we next investigated the VCS structure of *Irx3*^−/−^;Cx40^+/EGFP^ mice during the early postnatal period. At postnatal day 4 (P4), consistent with adult hearts, RBB was often absent in *Irx3*^−/−^;Cx40^+/EGFP^ mice, but not in *Irx3*^+/+^;Cx40^+/EGFP^ mice (Fig. 3a), in association with marked reductions in VCS density and thinner EGFP-positive fibers, particularly in the distal Purkinje fibers (Fig. 3b). On the other hand, at P0, only mild morphological defects were observed in *Irx3*^−/−^;Cx40^+/EGFP^ hearts compared with control mice. These findings suggest that loss of *Irx3* impacts on the postnatal development of the VCS. Consistent with this suggestion, quantification of the number of VCS fibers at three different levels (i.e. base, mid, and apex) revealed that *Irx3*^−/−^;Cx40^+/EGFP^ hearts exhibit only mild (~20%), but not significant, reductions in the number of fibers located in distal (i.e. mid and apex) VCS at P0, whereas by P4 (and thereafter) profound reductions (~50%; *P* < 0.02) in fiber numbers were observed throughout the VCS of *Irx3*^−/−^ hearts (Fig. 3c,d). Thus, VCS morphology appears to rapidly deteriorate beginning at birth in the absence of *Irx3*.

To determine whether this swift degradation of the VCS during the early postnatal period is caused by cell death or abnormal development, we measured cell apoptosis and proliferation in P4 *Irx3* mutant mouse hearts containing the Cx40-EGFP transgenic reporter. Consistent with the above observations, smaller EGFP-positive populations (i.e. the VCS) and weaker EGFP intensity were found in *Irx3*^−/−^;Cx40^+/EGFP^ hearts than *Irx3*^+/+^;Cx40^+/EGFP^ hearts (Fig. 3e). However, the number of cleaved caspase-3-positive cells were very low (less than 1 in 2500 cells) and indistinguishable between *Irx3*^+/+^;Cx40^+/EGFP^ and *Irx3*^−/−^;Cx40^+/EGFP^ hearts (Supplementary Fig. 5). On the other hand, the proliferation rate estimated by the total number of cells with phospho-histone H3 (PH3) expression was noticeably elevated in *Irx3*^−/−^;Cx40^+/EGFP^ hearts compared to WT controls (Fig. 3e and Supplementary Fig. 6). Specifically, the number of PH3-positive cells increased (*P* < 0.001) in the EGFP-negative populations (i.e. working myocardium; WM) of *Irx3*^−/−^ hearts when compared to the EGFP-positive populations (*P* = 0.104) (Fig. 3f). These data suggest that loss of *Irx3* leads to increased proliferation, which might disturb the cell cycle exit required for recruitment and differentiation of ventricular cardiomyocytes into mature VCS cells.

**Figure 4:**
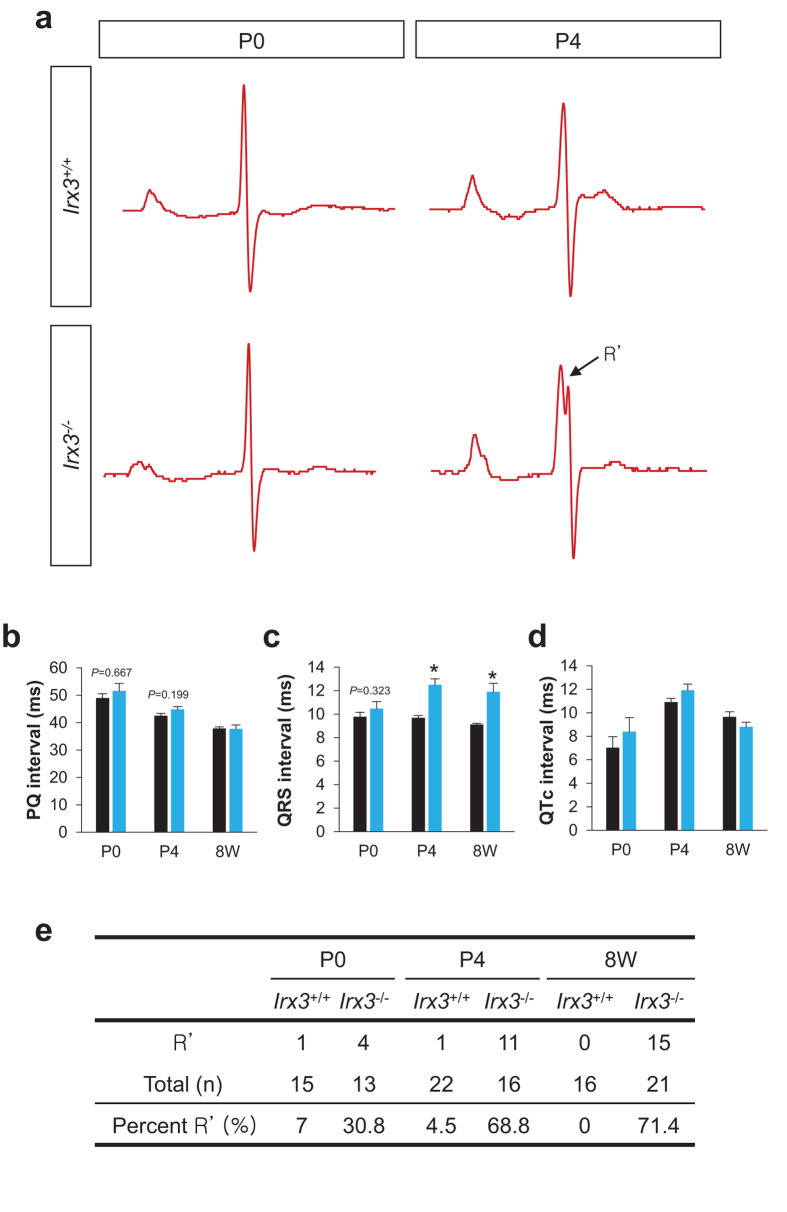
Postnatal electrophysiological defects in *Irx3*^−/−^ mice. (**a**) Representative ECG traces of *Irx3*^+/+^ and *Irx3*^−/−^ mice at P0 and P4. Analysis of neonatal mouse ECGs revealed that there are no differences in PQ (**b**) and QTc (**d**) intervals between *Irx3*^+/+^ and *Irx3*^−/−^ mice all different developmental stages (i.e. P0, P4 and 8 week old (8 W)). On the other hand, QRS intervals of *Irx3*^−/−^ mice, (**c**) that were not significantly different at P0 compared to controls, became prolonged from P4 onwards. (**e)** The prevalence of notched R’ wave, an indicator of right bundle branch block. *P < 0.05 vs. *Irx3*^+/+^ mice.

Next, we examined whether changes in the VCS structure were accompanied by electrophysiological changes. Surface ECG measurements revealed that PQ intervals, which progressively shorten with aging in both groups of mice, are not significantly different between *Irx3*^+/+^ and *Irx3*^−/−^ mice (Fig. 4a,b) at any time point (P0, P4, and 8 weeks old (8 W)). QRS durations, which reflect the time for activation of ventricles, are not different (*P* = 0.323) between *Irx3*^−/−^ mice (10.3 ± 0.5 ms) and WT littermate controls (9.7 ± 0.3 ms) at P0 (Fig. 4c), but are prolonged (*P* < 0.001) at P4 in the *Irx3*^−/−^ mice (12.4 ± 0.4 ms) compared to *Irx3*^+/+^ mice (9.8 ± 0.2 ms), which are similar to that in 8 week old adult *Irx3*^−/−^ mice (11.9 ± 0.7 ms). By contrast, QTc intervals are indistinguishable between *Irx3*^+/+^ and *Irx3*^−/−^ mice at all ages tested (Fig. 4d). Importantly, notching of R-waves, an indicator of RBBB, was rarely observed in WT controls, but was seen in 31% (4/13) at P0 and rose to 69% (11/16) by P4 in *Irx3*^−/−^ mice which is similar to the prevalence seen in adult *Irx3*^−/−^ mice (71.4%, 15/21) (Fig. 4e). These results establish that the electrophysiological changes in *Irx3*^−/−^ mice are accompanied by rapid progressive deterioration of VCS structure during the early postnatal period (within the first week after birth) and Irx3 is involved in early postnatal maturation of the VCS. Since the effects of *Irx3* ablation on the expression of Cx40 and Cx43 are first detected relatively early in the developmental period compared to the postnatal structural deterioration, the requirement of *Irx3* on gap junction expression appears temporally separated from that on the formation of the VCS structure.

### Molecular and functional associations of Irx3 with Nkx2.5 and Tbx5 in the heart

**Figure 5:**
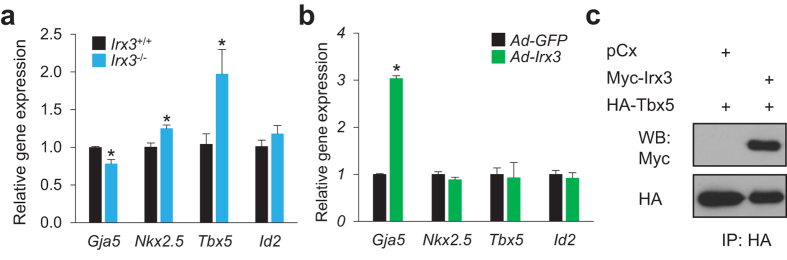
Irx3 physically interacts with Tbx5. (**a**) Gene expression analysis showed a reduction in Cx40 (*Gja5*) expression, but increases in *Nkx2.5* and *Tbx5* expression in P4 *Irx3*^−/−^ endomyocardium compared to controls. n = 5 for each group. *P < 0.05 vs. *Irx3*^+/+^ heart. (**b**) Adenovirus-mediated *Irx3* overexpression (*Ad-Irx3*) in cultured neonatal ventricular myocytes resulted in elevated Cx40 expression, but no changes in *Nkx2.5*, *Tbx5* and *Id2* expression, compared to control cells infected with *GFP*-expressing adenovirus (*Ad-GFP*). n = 3 for each group. *P < 0.05 vs. *Ad-GFP*. (**c**) Co-immunoprecipitation assay demonstrated a direct physical interaction of Irx3 with Tbx5.

Previous studies have established that Irx3 expression overlaps with the expression of the Nkx2.5, Tbx5 and Id2 transcription factors in the VCS, and that deficiencies of these transcription factors in mice (i.e. *Nkx2.5*^+/−^, *Tbx5*^+/−^, or *Id2*^−/−^) develop ventricular conduction defects with hypoplastic VCS morphology, widen QRS complex and/or reduced Cx40 expression^8^, which are remarkably similar to phenotypes observed in *Irx3*^−/−^ mice. It seems plausible that the overlapping phenotypes among these different mouse models may arise from genetic or protein-protein interactions. Since gene dosage of *Nkx2.5* and *Tbx5* are clearly critical for VCS morphology^8^, we performed gene expression analysis in micro-dissected left ventricular endomyocardium at P4. Cx40 (*Gja5*) gene expression is significantly reduced in P4 *Irx3*^−/−^ heart (Fig. 5a), as seen in adult *Irx3*^−/−^ heart^14^. On the other hand, we unexpectedly observed that *Nkx2.5* and *Tbx5* expression is up-regulated (*P* = 0.007 and 0.029, respectively) in the early postnatal period in the *Irx3*^−/−^ hearts compared to wild-type, which suggests that the VCS defects in *Irx3*^−/−^ heart do not result from reduced gene dosage of *Nkx2.5* or *Tbx5*. To examine further whether Irx3 directly regulates the expression of these cardiac TFs, *Irx3* was overexpressed in cultured neonatal mouse ventricular cardiomyocytes (NMVM), using adenoviruses. However, though *Irx3* overexpression led to elevated Cx40 gene (*Gja5*) expression, it did not alter the expression of *Nkx2.5, Tbx5* or *Id2* in the cultured cardiomyocytes (Fig. 5b), suggesting that elevations in *Nkx2.5* and *Tbx5* in P4 *Irx3*^−/−^ heart might represent a compensatory response. An alternative conceivable explanation for the common phenotypes seen in mice lacking *Irx3* and those heterozygous for *Nkx2–5* or *Tbx5* is that these transcription factors might interact directly in regulating VCS development. Indeed, we found that Irx3 and Tbx5 interact (Fig. 5c), as do Irx3 and Nkx2.5, which we showed previously^14^. These observations are consistent with the possibility that Irx3 interacts with Nkx2.5 and/or Tbx5 in a transcriptional complex that regulates the expression of genes required for VCS development and function.

**Figure 6:**
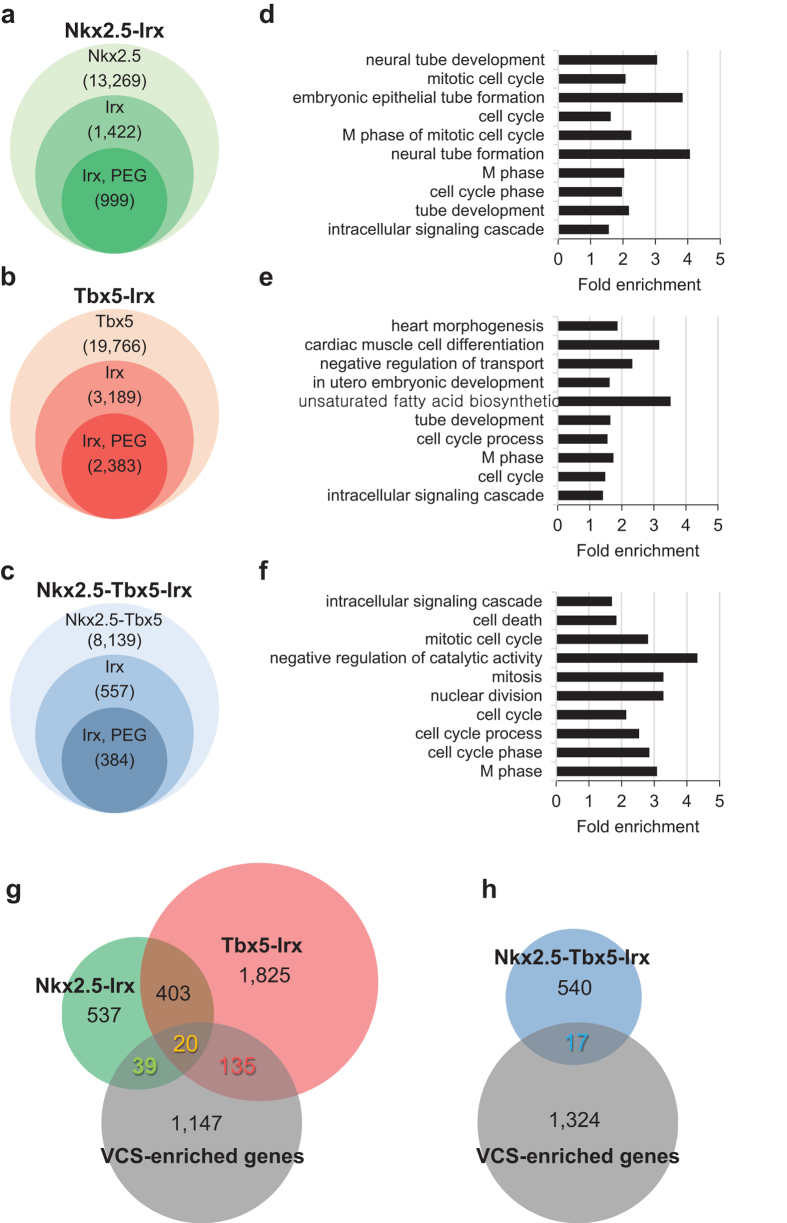
*In silico* analysis identifies Nkx2.5, Tbx5, and Irx3 target genes. (**a–c)** Venn diagrams showing the number of genes with ChIP-seq peaks, *Irx* motifs, and protein encoding genes (PEG) for Nkx2.5 (**a**), Tbx5 (**b**), and Nkx2.5-Tbx5 (**c**). (**d–f**) Selected GO terms associated with biological process for Nkx2.5-Irx (**d**) Tbx5-Irx (**e**) and Nkx2.5-Tbx5-Irx (**f**) genes (for complete data set, see Supplemental Dataset). (**g–h**) Venn diagrams showing the overlap and distribution of Nkx2.5-Irx (green) & Tbx5-Irx genes (red), as well as Nkx2.5-Tbx5-Irx genes (yellow or blue) in comparison with VCS-enriched genes.

In order to test whether Irx3 works together with Nkx2.5 and/or Tbx5 in the regulation of gene expression, we first performed genome-wide *in silico* analysis to identify their target genes by using available ChIP-seq datasets combined with motif-based sequence analysis with the MEME suite^21^ (Supplementary Fig. 7a). Specifically, from the ChIP-seq peak analysis of biotinylated Nkx2.5 and Tbx5 in HL-1 cardiac cell line^22^, we identified 13,269 and 19,766 genes containing Nkx2.5 and Tbx5 binding, respectively, within +/−50 kb of the transcription start site (TSS) (Fig. 6a,b). Next, to predict Irx3 target genes, we used the FIMO (find individual motif occurrence) program to scan genome-wide sequences with two putative *Irx* motif sequences, ‘ACATGT’^23^ and ‘ACAnnTGT’^24^ (Supplementary Fig. 7b). Analysis to assess the distribution of *Irx* motif sequences relative to Nkx2.5 or Tbx5 ChIP-seq peaks showed that *Irx* motifs were mainly enriched within 1000 bp of the Nkx2.5 or Tbx5 peaks (Supplementary Fig. 8), consistent with the hypothesis that Irx3 works closely with Nkx2.5 and/or Tbx5 during transcriptional regulation. As the distribution of distances between Nkx2.5 and Tbx5 peaks showed a peak at about 500 bp, we then applied more stringent criteria of selecting genes containing *Irx* motifs within 500 bp from either Nkx2.5 or Tbx5 peaks, identifying 1,422 genes for Nkx2.5-Irx and 3,189 genes for Tbx5-Irx co-occurrence (Fig. 6a,b and Supplementary dataset). We also examined the possible common targets of three transcription factors, and identified 557 genes as Nkx2.5-Tbx5-Irx co-occurrence among 8,139 genes containing both Nkx2.5 and Tbx5 peaks within 500 bp (Fig. 6c). To interpret Nkx2.5-Irx,Tbx5-Irx, or Nkx2.5-Tbx5-Irx downstream targets in biological process, we performed gene ontology (GO) term analysis only for protein encoding genes (PEG), which are 999, 2,383, and 384 genes for Nkx2.5-Irx, Tbx5-Irx, and Nkx2.5-Tbx5-Irx, respectively (Fig. 6d,e,f, and Supplementary dataset). Interestingly, cell cycle related functions were the top enriched GO terms in all Nkx2.5-Irx, Tbx5-Irx and Nkx2.5-Tbx5-Irx candidate genes.

To further narrow down and identify Nkx2.5-Irx3, Tbx5-Irx3 and Nkx2.5-Tbx5-Irx3 target genes, which are preferentially expressed in the VCS, we scanned the VCS-enriched genes (≥1.5 fold change in PF vs. WM) identified from microarray experiments using sorted cardiomyocytes isolated from adult Contactin-2-EGFP;αMHC-Cre;R26^tdTomato/+^ reporter mice^25^ (Supplementary dataset). This comparison revealed that 59 (green) and 155 (red) target genes of Nkx2.5-Irx and Tbx5-Irx groups, respectively, are enriched in the VCS (Fig. 6g). Among them, 20 genes (yellow) overlap in both Nkx2.5-Irx and Tbx5-Irx, consistent with previous studies that Nkx2.5 and Tbx5 cooperate in the VCS development^8^. More importantly, the comparison between VCS-enriched genes and 384 Nkx2.5-Tbx5-Irx target genes led to identification of 17 genes (blue), which are all found in the “overlapping” genes between Nkx2.5-Irx-PF and Tbx5-Irx-PF groups (Fig. 6h and Supplementary dataset). This result supports the possibility that Irx3 forms a transcription complex with Nkx2.5 and Tbx5 together.

**Figure 7:**
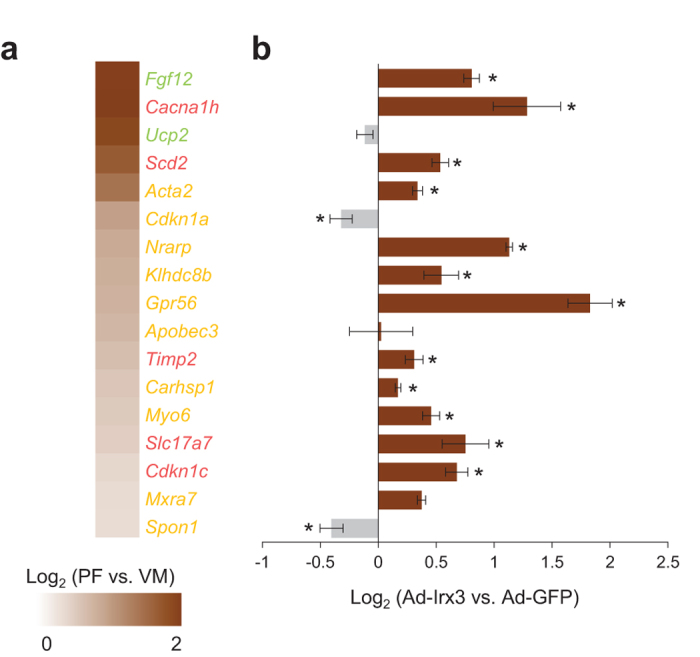
Irx3 promotes expression of VCS-enriched Nkx2.5 and Tbx5 target genes. (**a**) Expression of selected VCS-enriched genes, shown as the log2 expression ratio between Purkinje fibers (PF) and working myocardium (WM). Gene are color-coded for Nkx2-5-Irx-VCS (green), Tbx5-Irx-VCS (red), and Nkx2.5-Tbx5-Irx-VCS (yellow). (**b**) qPCR experiment demonstrates that Irx3 activates most of VCS-enriched genes in cardiomyocytes, shown as the log2 expression ratio between *Ad-Irx3* and *Ad-GFP*. n = 3–5 for each group. *P < 0.05 vs. *Ad-GFP*.

**Figure 8:**
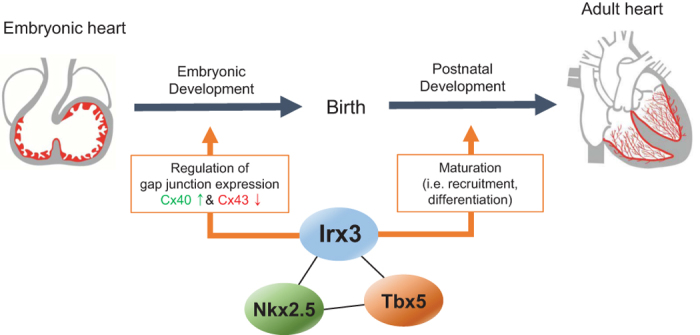
Irx3 is essential for VCS development and function. A schematic model of Irx3 cooperating with Nkx2.5 and Tbx5 in the VCS development and function.

To functionally validate whether these predicted target genes are regulated by Irx3, we tested top 10 candidate genes out of 17 VCS-enriched Nkx2.5-Tbx5-Irx genes, as well as several top candidate genes (≥2 fold change in VCS vs. WM) from Nkx2.5-Irx-PF and Tbx5-Irx-PF gene lists, such as *Fgf12*, *Ucp2*, *Cacna1h*, *Scd2*, *Timp2*, *Slc17a7*, and *Cdkn1c* (Fig. 7a). qPCR analysis in NMVM overexpressing *Irx3* showed that Irx3 promotes significant upregulation in most of candidate genes, including *Fgf12*, *Cacna1h*, *Nrarp*, *Gpr56*, *Myo6*, *Slc17a7*, *Cdkn1c* and *Mxra7* (Fig. 7b). Thus, our *in silico* analysis coupled with functional verification suggests that Irx3 acts as a co-factor of Nkx2.5 and/or Tbx5 in transcriptional regulation of VCS genes. Taken together, our current and previous studies^14^ suggest a model wherein Irx3 interacts with Nkx2.5 and/or Tbx5 in a transcriptional complex required for proper development and function of the VCS (Fig. 8), and loss of *Irx3* might lead to disrupted Nkx2.5 and/or Tbx5 transcriptional complexes resulting in VCS abnormalities seen similarly in *Nkx2.5* or *Tbx5* mutant mice.

## Discussion

We previously showed that loss of *Irx3* is associated with impaired conduction in the VCS, such as slowed ventricular activation and RBB block^14^. These studies further revealed that *Irx3* ablation leads to reduced Cx40 expression in the VCS combined with ectopic Cx43 expression in the proximal bundle branches, consistent with molecular studies establishing that Irx3 antithetically regulates the gap junction genes, Cx40 (*Gja5*) and Cx43 (*Gja1*), in the VCS^14^. However, several observations suggest that the electrical changes in *Irx3*^−/−^ mice could also involve other factors as well. For example, although Cx40^+/EGFP^ and *Irx3*^−/−^ mice show similar (~2-fold) reductions in Cx40^18^, Cx40 heterozygous mutant mice do not show overt cardiac conduction defects, which suggest that *Irx3* may also have other effects on the VCS, in addition to its actions on gap junction expression. Accordingly, in this study we demonstrate that Irx3 plays an essential role in maintaining the structural integrity of the VCS in the postnatal period. Specifically, loss of *Irx3* results in progressive deterioration of the His-Purkinje system, which begins at birth. The profound reductions in the fiber density are expected to impair cardiac electrical activation leading to widening of the QRS interval and the RBB block that are seen in *Irx3*^−/−^ mice. The appearance of RBB block, without evidence for LBB block, is not unexpected due to the dramatic differences in the structures of the right and left bundle branches. Specifically, the left bundle branches have an abundance of fascicles compared to the right bundle branches which often has only one or two strand in both mice and human^16^,^26^. As a consequence of reductions in the right and left bundle branches arising with the loss of *Irx3*, we often observed the absence of right bundle branches, which would explain the appearance of RBB block.

It is worth mentioning that the structural defects of the VCS and altered gap junction expression in *Irx3*^−/−^ heart appear to be temporally discordant, suggesting that they may not be inter-dependent. First, at E15.5, when the Cx40 and Cx43 expression has already restricted to the VCS and the working myocardium^5^,^20^, respectively, *Irx3*^−/−^ hearts show a normal morphology of fiber structure, despite ectopic expression of Cx43. Second, mice lacking Cx40, which show almost identical electrophysiological defects to *Irx3*^−/−^ mice, have normal structure of VCS. Third, morphological defects in *Irx3*^−/−^ VCS are found prominently in regions where Cx43 is not ectopically expressed (i.e. distal Purkinje fibers). Nevertheless, there is a possibility that ectopic Cx43 expression and reduced Cx40 expression *Irx3*^−/−^ embryonic hearts, which are expected to functionally affect the embryonic heart pumping, can influence on postnatal maturation of the VCS structure, as previous studies show that hemodynamics can affect VCS development and maturation^27^,^28^.

Our results revealed that Irx3 is essential for postnatal development of the VCS, since the number and thickness of the His-Purkinje fiber network as well as the rate of ventricular activation (i.e. QRS intervals) are reduced progressively after birth. Previous studies suggest that VCS development can be largely divided into two temporal stages^12^. First, a prenatal VCS developmental process involves expansion of pre-specified conduction cells as well as recruitment and expansion of cells derived from the underlying trabeculae of the embryonic ventricle^1^. Then, during postnatal period, the VCS is further differentiated, resulting in the formation of the mature Purkinje fiber network. While no noticeable changes in VCS structure or trabecular layer were observed in embryonic *Irx3*^−/−^ hearts at E15.5, there were elevated numbers of proliferating cells in postnatal *Irx3*^−/−^ heart (i.e. P4). These findings indicate that postnatal VCS defects in *Irx3*^−/−^ heart are probably not caused by abnormal trabeculation during embryonic development, but by a postnatal maturation defect, possibly due to delay cell cycle exit after birth. Indeed, increased proliferation was seen in surrounding (i.e. Cx40-negative) working myocardium of *Irx3*^−/−^ heart without changes in apoptotic death of VCS cells. These are further supported by our computational analysis showing that predicted Nkx2.5-Irx, Tbx5-Irx and Nkx2.5-Tbx5-Irx target genes are enriched for cell cycle-associated function as well as by the functional verification showing that Irx3 altered transcript levels of *Cdkn1a* and *Cdkn1c*, which encode cyclin-dependent kinase inhibitors, p21 and p57, respectively, yet how these function during VCS development and maturation remains to be explored. In addition, it has been recently reported that Drosophila *Iroquois* proteins restrict cell cycle progression via non-transcriptional mechanism with a physical interaction with Cyclin E-containing protein complexes^29^. Together, ours and other studies suggest a possibility that Irx3 controls cell cycle, thereby regulating postnatal recruitment and differentiation into mature VCS cells from progenitor cells residing in working myocardium. Previous studies have demonstrated that expansion and recruitment of VCS cells from nearby working ventricular cardiomyocytes are positively affected by endothelin-1^30^,^31^ and neuregulin-1^32^ during embryonic heart development. Therefore, it will be interesting to determine the mechanism by which Irx3-expressing VCS cells regulates the recruitment and differentiation of Irx3-negative working myocardium into mature VCS cells during postnatal development.

It is notable that the hypoplastic VCS defects observed in *Irx3*^−/−^ mice are remarkably similar to those seen in mice with haploinsufficiency of *Nkx2–5*^4^,^12^ and *Tbx5*^7^. Indeed, our results showing a direct interaction of Irx3 with Tbx5 as well as Nkx2.5 strongly support a model that a transcriptional complex of these factors is critical for development and maintenance of the VCS structure and function. This model is supported by bioinformatic predictions followed by the functional verification demonstrating that Irx3 functions as a transcriptional regulator on the genes to which Nkx2.5 and/or Tbx5 bind.

While our *in silico* analysis combined with qPCR verification using Irx3-overexpressed cardiomyocytes identified candidate target genes of Nkx2.5, Tbx5 and Irx3, there is still limitation to conclude whether this is through direct or indirect regulation, due to complexity of gene regulation. For example, it was unexpected that Cx40 gene, *Gja5*, which we and other have demonstrated that Irx3 positively regulates its expression^14^,^15^, was not found via our bioinformatics approaches. As shown in the Supplementary Fig. 9a, *Gja5* contains co-occurrence of Nkx2.5 and Tbx5 ChIP-seq peaks as well as *Irx* motif which is located about 1 kb away (that is why our stringent method eliminated *Gja5* from the candidate list), yet these peaks and motifs are all found, not in the promoter region, but at around 10 kb away from the transcriptional end site. In addition, we found that, in most of top candidate genes, Nkx2.5 and Tbx5 ChIP-seq peaks with *Irx* motif were found in downstream of the TSS (i.e. intron) or extragenic region (Supplementary Fig. 9b and Supplementary dataset). As discussed previously^33^, our observations suggest that Irx3 may be involved in complex transcriptional regulation via genetic elements such as enhancer. Therefore, in order to further decipher the mechanistic details of transcriptional regulation by Irx3, Nkx2.5 and Tbx5, additional experiments using various techniques, such as ChIP-seq for Irx3 and other epigenetic marks, chromosome conformation capture, a reporter assay will be necessary. Also, as Irx3 has dual functions depending on developmental stages, it would be of interest to delineate embryonic and postnatal transcriptional regulatory networks within VCS. Interestingly, most of 17 candidate target genes of Nkx2.5-Tbx5-Irx3 from the bioinformatics analysis have not been previously associated with the VCS or even with cardiovascular system, as summarized in Supplementary dataset. Therefore, future studies are warranted to examine whether/how these genes contribute to VCS development and function.

Together, in addition to our previous findings^14^, this study revealed a novel role for *Irx3* in the morphological development of the VCS, demonstrating that Irx3 is a conductor of ventricular conduction system^34^ by maintaining the integrity of VCS structure and function. As mutations in *NKX2-5*^35^,^36^,^37^ and *TBX5*^38^,^39^,^40^ are found in patients with conduction defects, a recent study has identified two novel *IRX3* mutations, R421P and P485T, in idiopathic VF patients without other genetic defects in well-known arrhythmogenic genes^15^. *In vitro* experiments also showed that mutated forms of *IRX3* led to reduced transcriptional regulation compared to wild-type *IRX3*, suggesting that these mutant *IRX3* alleles associated with arrhythmia act as hypomorphic or loss-of-function mutations. Therefore, as anatomy and development of the VCS is well conserved between mouse and human, our findings here would provide additional insight and mechanism for conduction defects associated with *IRX3* mutations.

## Methods

### Animals

All animal experiments were performed in accordance with protocols approved by The Faculty of Medicine and Pharmacy Animal Care Committee, University of Toronto as well as by The Toronto Centre for Phenogenomics Animal Care Committee (ACC), and conformed to the standards of the *Canadian Council on Animal Care*. All mice were housed in standard vented cages in temperature- and humidity-controlled rooms with 12-hour light-dark cycles. *Irx3*^+/−^ and *Irx3*^+/LacZ^ mice were previously described^14^, and were maintained in the CD1 background. Neonatal and adult Irx3 mice were generated by intercrossing *Irx3*^+/−^ mice. In order to visualize the VCS, *Irx3*^+/−^ mice were crossed with Cx40^+/EGFP^ knock-in reporter mice that express GFP under the control of the *Gja5* (Cx40) promoter^16^ (courtesy of Dr. Lucile Miquerol). For comparison of VCS morphology using β-galactosidase staining, *Irx3*^+/LacZ^ mice were intercrossed with *Irx3*^+/−^ mice to generate *Irx3*^+/LacZ^ and *Irx3*^−/LacZ^ mice, which allow us to properly compare control (i.e. heterozygous) and knock-out mouse hearts under single copy of *LacZ* gene.

### ECG measurements

Mice were anesthetized using a mixture of isoflourane and oxygen. Anesthetized mice were secured in a supine position on a regulated heat pad while lead I and lead II ECGs were recorded using platinum subdermal needle electrodes in a 3-limb configuration. Core temperature was continuously monitored using a rectal probe and maintained at 36–37 °C. ECG data was acquired and analyzed using PONEMAH Physiology Platform P3 Plus software and an ACQ-7700 acquisition interface unit (Gould Instruments, Valley View, OH, USA). The parameters derived from the ECG measurements include: Heart rate (HR), PR interval (beginning of P wave to the beginning of the QRS complex), QRS complex duration, and QT interval (beginning of Q wave to end of T wave).

### Optical mapping and analysis

Mice were heparinized (0.2 ml intraperitoneal injection (I.P.) of 1000 IU/ml heparin) to prevent blood clotting and were sacrificed under isofluorane via cervical dislocation. The thorax was opened by midsternal incision, and the beating heart was rapidly removed and placed in cold Krebs solution consisting of (in mmol/L) 118 NaCl, 4.2 KCl, 1.2 KH_2_PO_4_, 1.5 CaCl_2_, 1.2 MgSO_4_, 11 D-Glucose, 2 Sodium Pyruvate, and 25 NaHCO_3_, with pH adjusted to 7.4 via bubbling with carbogen (95% CO_2_/5% O_2_). The heart was then rapidly transferred to the bath of a modified horizontal Langendorff in cold Krebs solution where the aorta was cannulated onto a blunted 20-gauge needle attached to a bubble trap and a water jacket-heated perfusion system to allow for retrograde perfusion of the coronary arteries while enabling the rotation of the heart about its vertical axis. Once mounted, the heart was perfused with Krebs solution at a constant flow rate of 3.5 ml/min, for a 15 minute equilibration period followed by 10 minute of voltage sensitive dye, di-4-ANEPPS, perfusion and wash. A concentration of 0.1 μM ATP dependent K^+^ channel opener, P1075, was added to the perfusing Krebs solution in order to offset the coronary constriction and rise in aortic pressure observed during dye administration^41^,^42^. Perfusion rates were maintained such that a coronary pressure of 70–90 mmHg was observed during measurements. Perfusion temperature was monitored and held at 36–37 °C throughout the experiment. All mapping studies were performed in the absence of any motion reduction techniques.

### Ventricular conduction system morphology: Fiber imaging, quantification of EGFP fluorescence, and measurement of fiber fraction

*Irx3*^+*/*+^;Cx40^+/EGFP^ and *Irx3*^−/−^;Cx40^+/EGFP^ adult (10–12 week old), P4 and P0 mice were anaesthetized and sacrificed via cervical dislocation. The hearts were rapidly excised and placed in room temperature standard PBS solution (pH 7.4). For imaging left ventricular conduction system, LV wall was carefully cut open at the centre of the free wall in adult, P4 and P0 mouse hearts, while making sure no major arteries or conduction fibers were damaged. The heart was pinned down and occasionally flattened with a glass slide to maximize the number of fibers in focus. Images of the hearts were taken using an upright Olympus MVX-10 microscope equipped with a CoolSnap HQ (Photometrics, Surrey, BC) camera interfaced with ImagePro Plus 5.1 software. *Irx3* deficient, heterozygous and wild-type mice with the Cx40-EGFP reporter were excited (EXFO X-Cute exacte). For comparison purposes, littermates were images on the same day at comparable magnification, exposure and light intensity. Cx40 promoter-dependent GFP expression was determined by measuring the intensity of EGFP fluorescence in the LBB and distal Purkinje fibers of *Irx3*^−/−^;Cx40^+/EGFP^, *Irx3*^+/−^;Cx40^+/EGFP^ and *Irx3*^+*/*+^;Cx40^+/EGFP^ mice. For control purposes, EGFP fluorescence intensity was also measured in the atria and septal artery of all three mouse groups, where *Irx3* is not expressed. Intensity of fluorescence was measured using Image J software in multiple fibers divided by the fiber area and averaged to obtain a value for the LBB and Purkinje fibers. To examine thickness and numbers of the VCS, fiber fraction was measured in left ventricular VCS of wild-type and *Irx3*^−/−^ heart with the background of Cx40-EGFP at P0 and P4. In brief, threshold was determined by measuring the level of background from 12 different regions containing myocardium without fiber using Image J. The left ventricular conduction system of all hearts was divided into 3 sections from the His bundle to the end of the distal Purkinje fibers (marked by dashed lines in Fig. 3c). Then, the number of pixels above threshold (>0; signifying pixels that account for fibers) along the three lines was counted.

### *LacZ* staining

*Irx3*^+/LacZ^ and *Irx3*^−/LacZ^ hearts were dissected in cold PBS, and fixed in 1% formaldehyde, 0.02% NP-40 in PBS. After fixation, hearts were washed in 2 mM MgCl_2_, 0.02% NP-40 in PBS at 4 °C for four times for 15 minutes each. For embryonic VCS examination, dissected E15.5 embryos were immediately frozen in OCT on dry ice. After cryo-sections taken at 25 μm, sections were fixed in 0.2% glutaraldehyde in PBS on ice for 10 minutes, and washed in detergent rinse for 10 min. For β-gal staining, hearts or slides were incubated in X-gal or Bluo-gal solution containing 1 mg/ml X-gal or Bluo-gal, 2 mM MgCl_2_, 0.02% NP-40, 5 mM K_4_Fe(CN)_6_-3 H_2_O, 5 mM K_3_Fe(CN)_6_ in PBS at 37 °C overnight. Following incubation, hearts or slides were fixed in 4% formaldehyde. Sections were further counterstained with Nuclear Fast Red (Vector Labs) for 1 minute and mounted with VectaMount AQ (Vector Labs).

### Immunofluorescent staining

P4 heart and E15.5 embryos were cut into 15–20 μm thick sections with a cryo stat. Sections were fixed with ice-cold acetone for 5 minutes, blocked with 5% normal donkey serum diluted in PBST for 1 hour at room temperature and incubated overnight with primary antibodies at 4 °C. After rinsing 3 times for 5 minutes with PBS, proper secondary antibodies (1:500) were incubated for 1 hour at room temperature. Sections were then rinsed 3 times with PBS and then mounted with DAPI-containing mounting medium (Vector Labs). The following primary antibodies were sued: anti-phosphor-histone H3 (rabbit, 1:200, Cell Signaling Technology), anti-cleaved caspase 3 (rabbit, 1:200, Cell Signaling Technology), anti-Cx40 (goat, 1:100, Santa Cruz Biotechnology), and anti-Cx43 (rabbit, 1:100, Cell Signaling Technology).

### Co-immunoprecipitation and immunoblotting

Co-immunoprecipitation was conducted in COS-7 cells 48 hours after transfection with PCx, Myc-Irx3, and HA-Tbx5 constructs, as previously described^14^. After incubation with the indicated antibodies overnight at 4 °C, immunoprecipitates were pulled down with protein G–Sepharose beads and immunoblotted with the indicated antibodies overnight at 4 °C, following standard protocols.

### Gene expression analysis

Total RNA was extracted from micro-dissected endomyocardium at P4 or cultured neonatal cardiomyocytes isolated^43^ and infected with adenovirus expressing GFP or Irx3, using TRIzol (Life Technologies), and then cDNA was synthesized with MMLV-RT (Life Technologies). Quantitative PCR was performed with Power SYBR green enzyme on Viia7 (Life Technologies), and ∆∆CT normalized with *Actb* or *Tbp* expression were used for relative comparison. Primer sequences are available upon request.

### Computational analysis and prediction of Irx3, Nkx2.5 and Tbx5 target genes

Two putative *Irx* motif sequences (Supplementary Fig. 7b), derived from ‘ACATGT’^23^ and ‘ACAnnTGT’^24^, and MotifMap database^44^, were used for predicting genome-wide *Irx3* motif occurrences. This was accomplished following a whole genome search of the putative motifs on mouse genome assembly GRCm38 sequence using FIMO (Find Individual Motif Occurrences), part of the MEME software package (http://tools.genouest.org/tools/meme/)^45^. Only motif occurrences with p-values less than or equal to 1.0 × 10^−5^ were considered. P-values are calculated by FIMO program and are based on a log-likelihood ratio score conversion. For more details of FIMO program calculations please refer to^46^.

To identify downstream genes of Nkx2.5, Tbx5 and Irx3 in the heart, we used previously described parameters for cardiac transcription factor binding sites proximity^22^ to define co-occurrence of either Nkx2.5 or Tbx5 peaks with *Irx* motifs near gene loci. Previously identified enriched Chromatin Immuno-precipitation sequencing (ChIP-seq) peak files for Nkx2.5 (GSM558906) and Tbx5 (GSM558908) (GEO accession # GSE21529) were downloaded in GFF format (as mm9 assembly coordinates). Their genomic coordinates were converted to GRCm38 sequence assembly coordinates using LiftOver tool (https://genome.ucsc.edu/cgi-bin/hgLiftOver) for co-occurrence analysis. Briefly, we first determined potential target genes by mapping Nkx2.5 and Tbx5 enriched peaks to the nearest genes within +/−50 kb of transcriptional start sites (TSS) as defined by Ensembl GRCm38 gene annotations. Gene loci associated with either Nkx2.5 or Tbx5 were then further examined for the presence of Irx motifs that are 500 bp or less away from Nkx2.5 and/or Tbx5 peaks. The DAVID software was used for Gene Ontology analysis on protein coding genes.

We further narrowed down the list of predicted target genes with regards to the VCS by comparing with VCS-enriched genes. List of VCS-enriched genes were obtained using the microarray dataset (GSE60987) that was performed in two populations of sorted cardiomyocytes (PF: Tomato^+^EGFP^+^ vs. WM: Tomato^+^EGFP^−^) isolated from the ventricles of adult compound transgenic mice expressing PF (Cntn2-EGFP) and cardiomyocyte specific (α-MHC–Cre;R26^tdTomato/+^) reporter genes^25^. Microarray data analysis was performed using Expression Console and Transcriptome Analysis Console software (Affymetrix) with criteria of ANOVA P-value < 0.05 and ≥1.5 fold changes.

### Statistical analysis

All results are expressed as mean ± S.E.M. (the standard error of the mean). Significance was determined by student *t*-test. Differences at *P* < 0.05 were considered statistically significant. Calculations and statistical test were performed using the Sigma Stat 3.0 program.

## Additional Information

**How to cite this article**: Kim, K.-H. *et al.* *Irx3* is required for postnatal maturation of the mouse ventricular conduction system. *Sci. Rep.* **6**, 19197; doi: 10.1038/srep19197 (2016).

## Supplementary Material

Supplementary Information

Supplementary Dataset
