# Supplementary material for: Irx3 is required for postnatal maturation of the mouse ventricular conduction system
Source: Sci Rep. 2016 Jan 20;6:19197. doi: 10.1038/srep19197 (PMC4726432; doi:10.1038/srep19197)

Supplementary information

Irx3 is required for postnatal maturation of the mouse ventricular conduction system

by

Kyoung-Han Kim[1,*], Anna Rosen[2,3,*], Samer M.I. Hussein[4, 5], Vijitha Puviindran[1], Adam S. Korogyi[2,3], Carmelina Chiarello[2,3], Andras Nagy[4, 6], Chi-chung Hui[1,7,#], Peter H. Backx[2,3,8,#]

Supplementary Figure Legends

Supplementary Figure 1 | Loss of *Irx3* leads to reduced fluorescent intensity of Cx40 promoter-driven EGFP in the VCS. Quantification of Cx40-dependent EGFP fluorescence intensity in the atria, septal artery, LBB and distal Purkinje fibers of 10-12 week old, *Irx3*$^{+/-}$;Cx40$^{+/EGFP}$ and *Irx3*$^{-/-}$;Cx40$^{+/EGFP}$ mice as a percent change of *Irx3*$^{+/+}$;Cx40$^{+/EGFP}$ mice. Values are mean ± S.E.M., n = 3.

Supplementary Figure 2 | Loss of *Irx3* leads to abnormal right bundle branch structure. In severe cases, right bundle branch was completely absent in 10-12 week old adult *Irx3*$^{-/LacZ}$ heart.

Supplementary Figure 3 | Prenatal VCS structures are indistinguishable between *Irx3*$^{+/LacZ}$ and *Irx3*$^{LacZ/LacZ}$ embryos. LacZ staining to mark Irx3-positive VCS revealed that there were no visible morphological defects of the VCS in embryonic heart lacking *Irx3* at E15.5, compared to control embryos.

Supplementary Figure 4 | Irx3 possesses a regulatory function of Cx43 expression in the proximal bundle branch from E15.5 onwards. Representative Cx43 and Cx40 immunofluorescence images in *Irx3*$^{-/-}$ embryonic heart at E15.5. Arrowheads show ectopic Cx43-positive plaques in Cx40-positive developing bundle branches.

Supplementary Figure 5 | VCS morphological defects in *Irx3*$^{-/-}$ heart is not mediated by cell death. Representative immunofluorescent images of cleaved caspase-3 at P4 heart. Low numbers of cleaved caspase-3-positive cells (less than 1 in 2500 cells) were observed in both *Irx3*$^{+/+}$;Cx40$^{+/EGFP}$ and *Irx3*$^{-/-}$;Cx40$^{+/EGFP}$ hearts without significant differences.

Supplementary Figure 6 | *Irx3*-deficient postnatal heart shows abnormal cell proliferation. Representative immunofluorescent images of phospho-histone H3 (PH3)-positive cells in the right ventricle of P4 heart. *Irx3*$^{-/-}$;Cx40$^{+/EGFP}$ hearts showed noticeably higher number of PH3-positive cells mainly in EGFP-negative myocardium, compared to *Irx3*$^{+/+}$;Cx40$^{+/EGFP}$ heart.

Supplementary Figure 7 | Computational analysis for Nkx2.5-Irx3 and Tbx5-Irx3 target genes. **a,** Candidate gene identification scheme. **b,** Irx TF binding motifs used for motif-based sequence analysis with FIMO. **c,** Distribution of distance between Irx motifs and ChIP-seq peaks of Nkx2.5 and Tbx5.

Supplementary Figure 8 | Distribution of distance between Irx motifs and ChIP-seq peaks of Nkx2.5 and Tbx5.

Supplementary Figure 9 | Alignments of Nkx2.5-Tbx5-Irx target gene dataset. *Gja5* (**a**) and Gpr56 (**b**) contain ChIP-seq peaks of Nkx2.5 and Tbx5 as well as Irx motif located around 10kb away from TES or in the intronic region, respectively.

Supplementary Figure 1

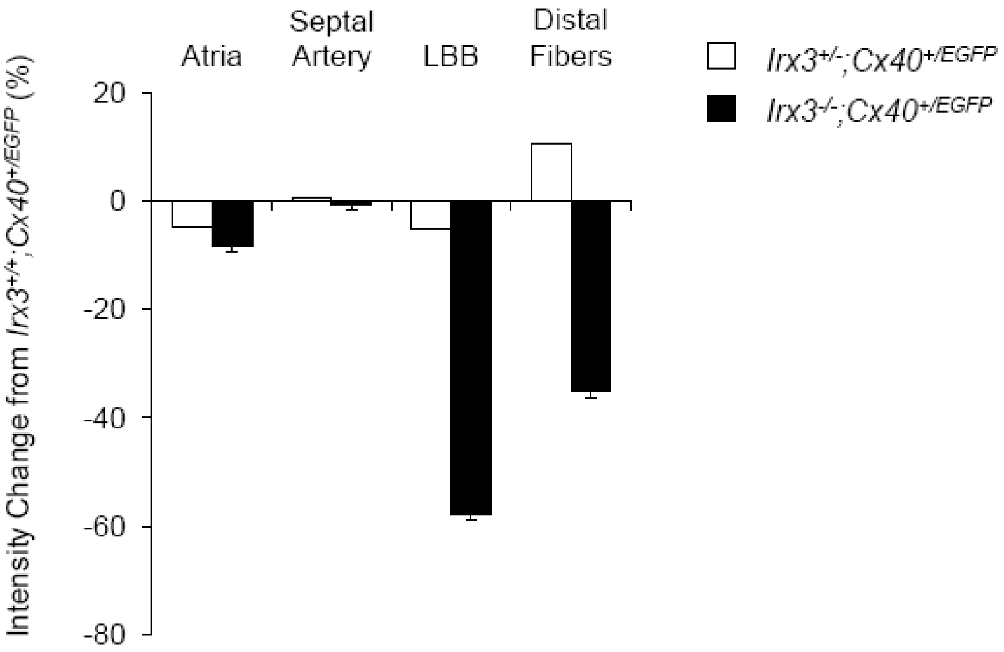

Supplementary Figure 2

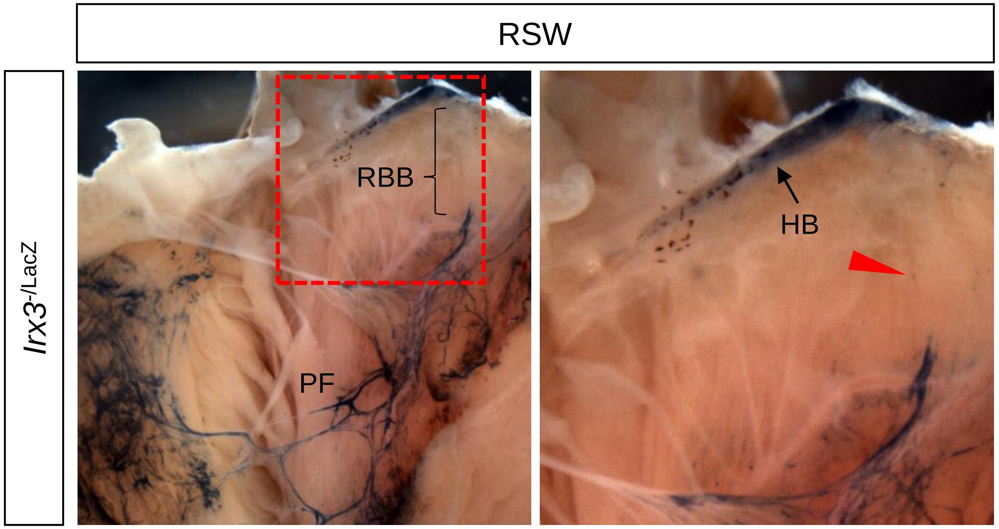

Supplementary Figure 3

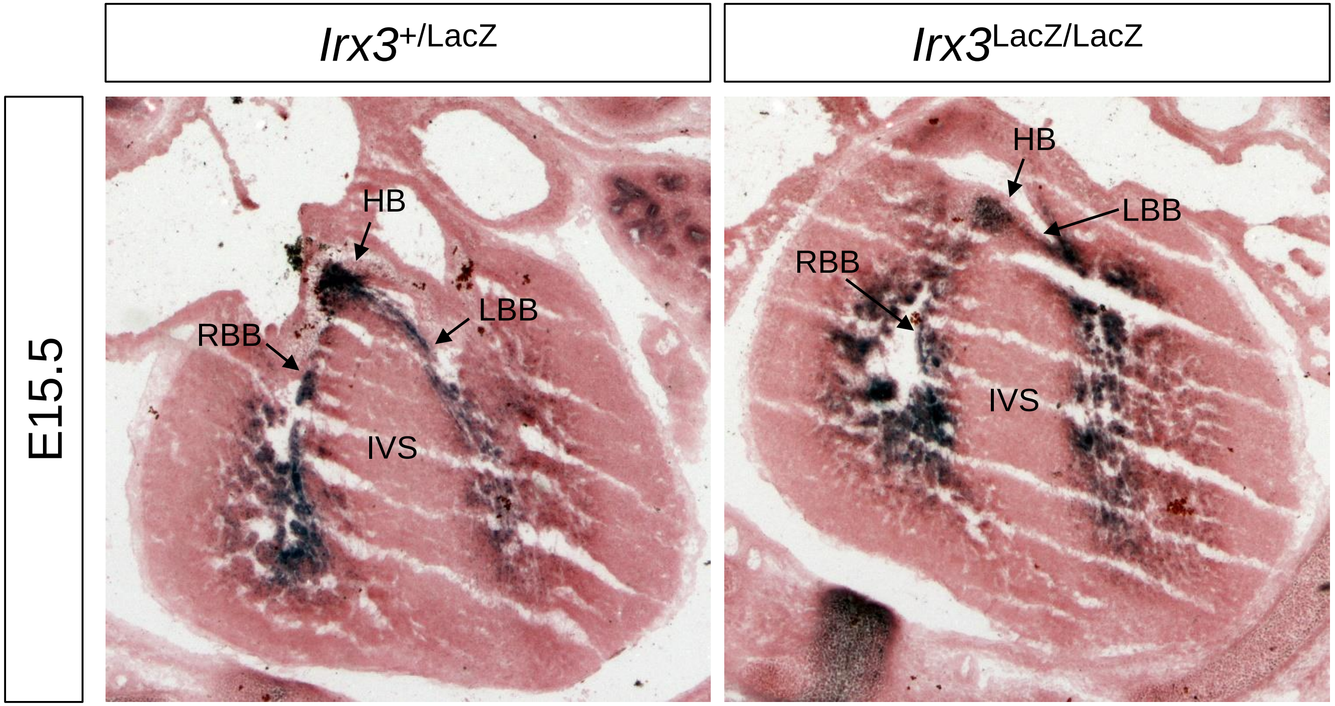

Supplementary Figure 4

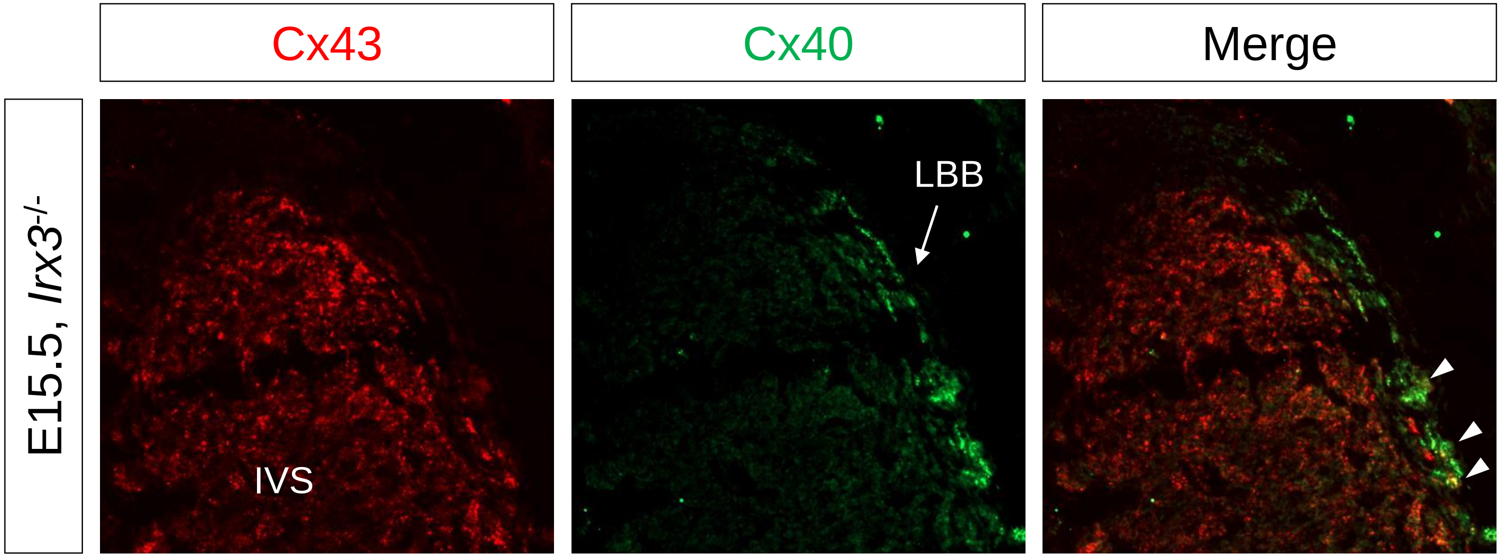

Supplementary Figure 5

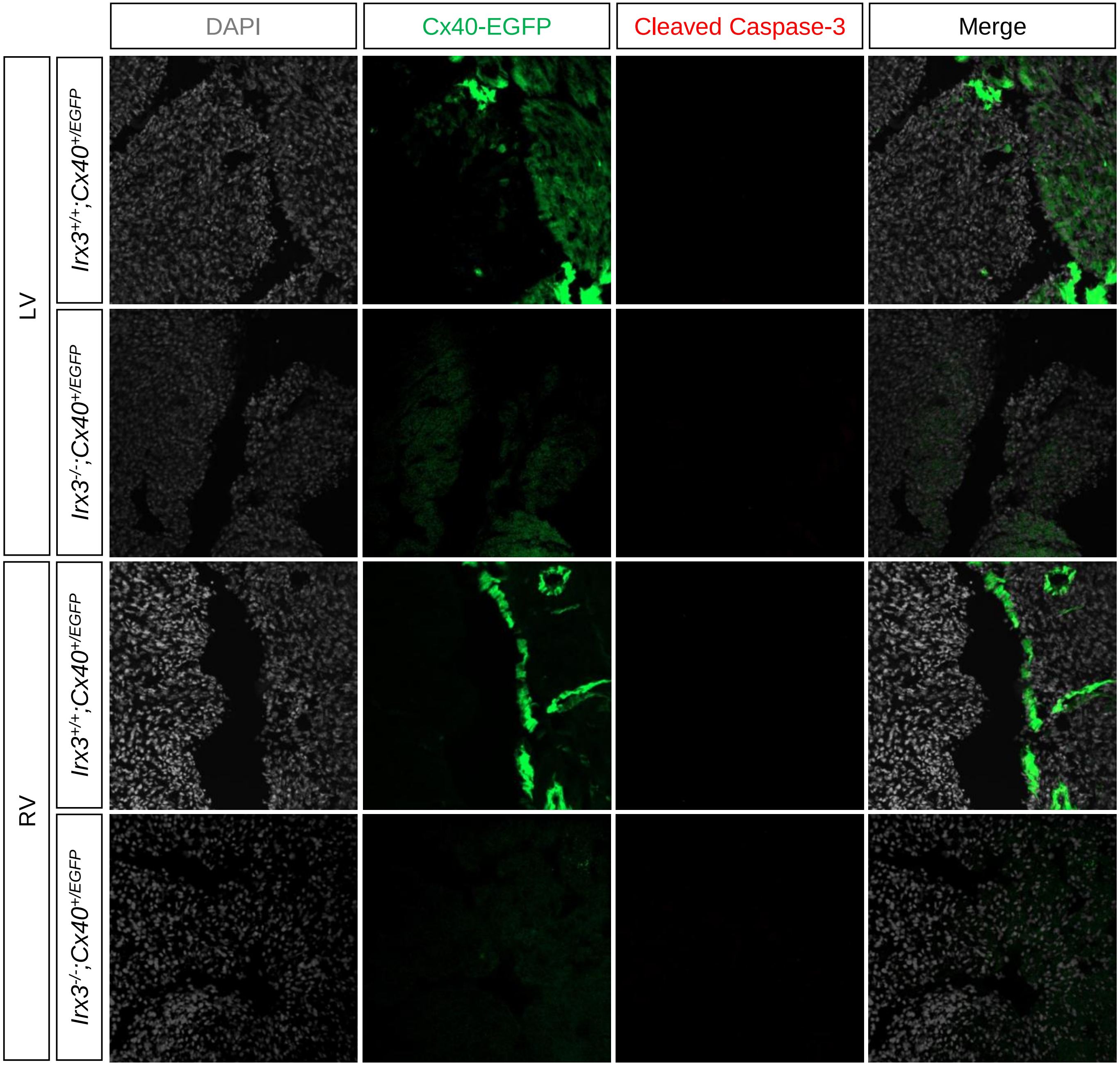

Supplementary Figure 6

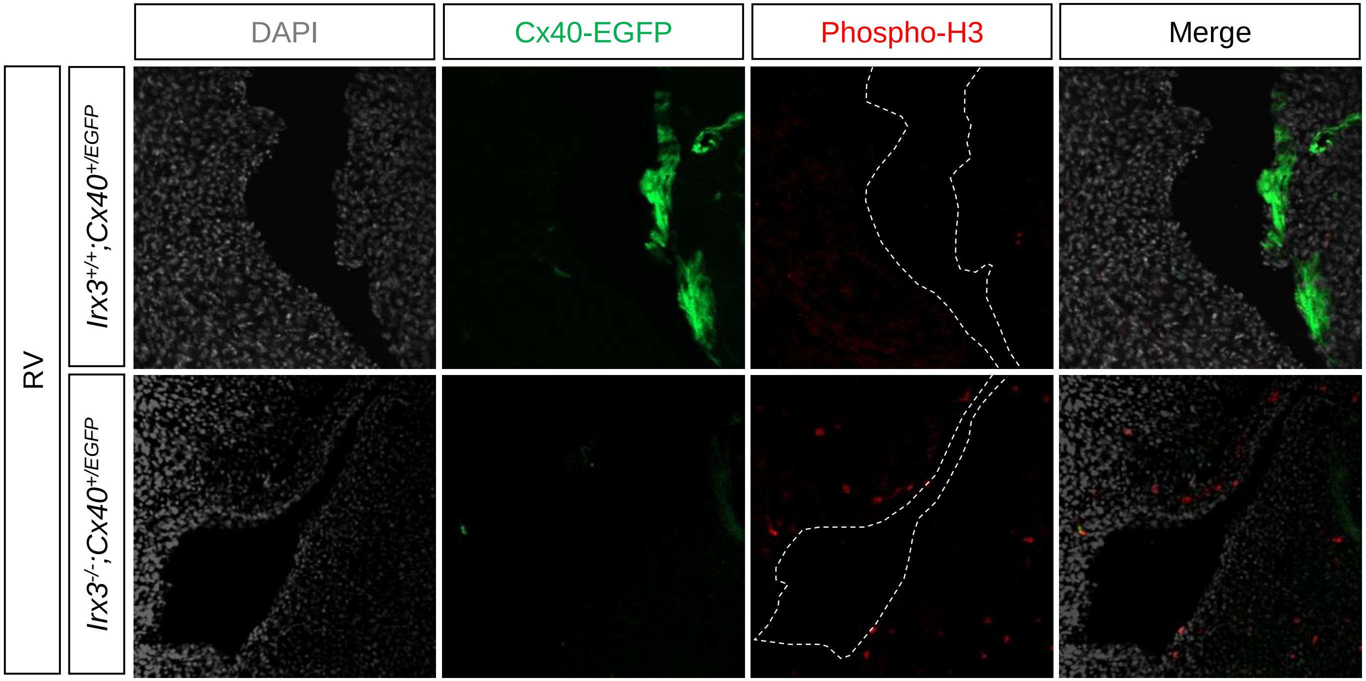

Supplementary Figure 7

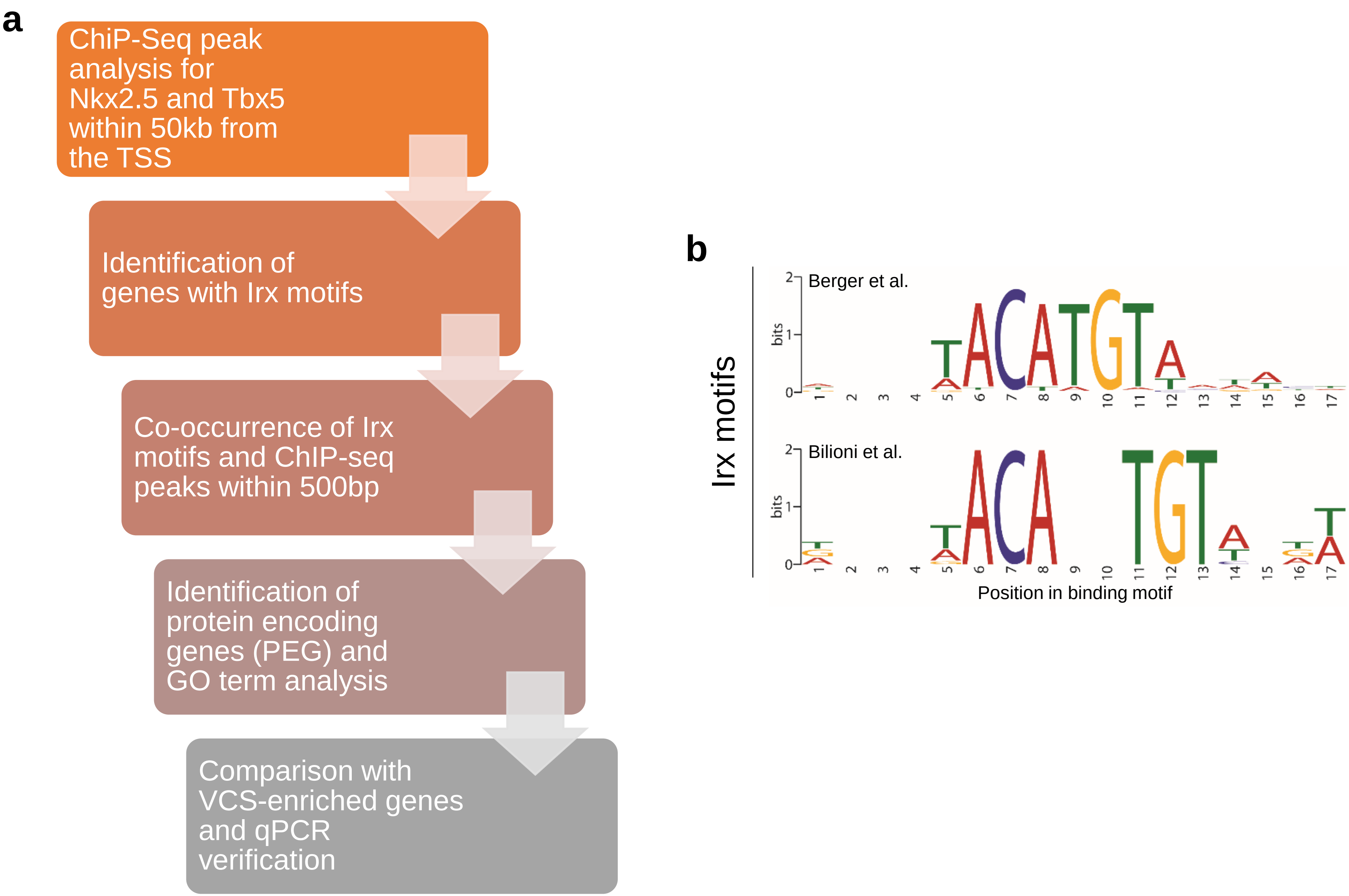

Supplementary Figure 8

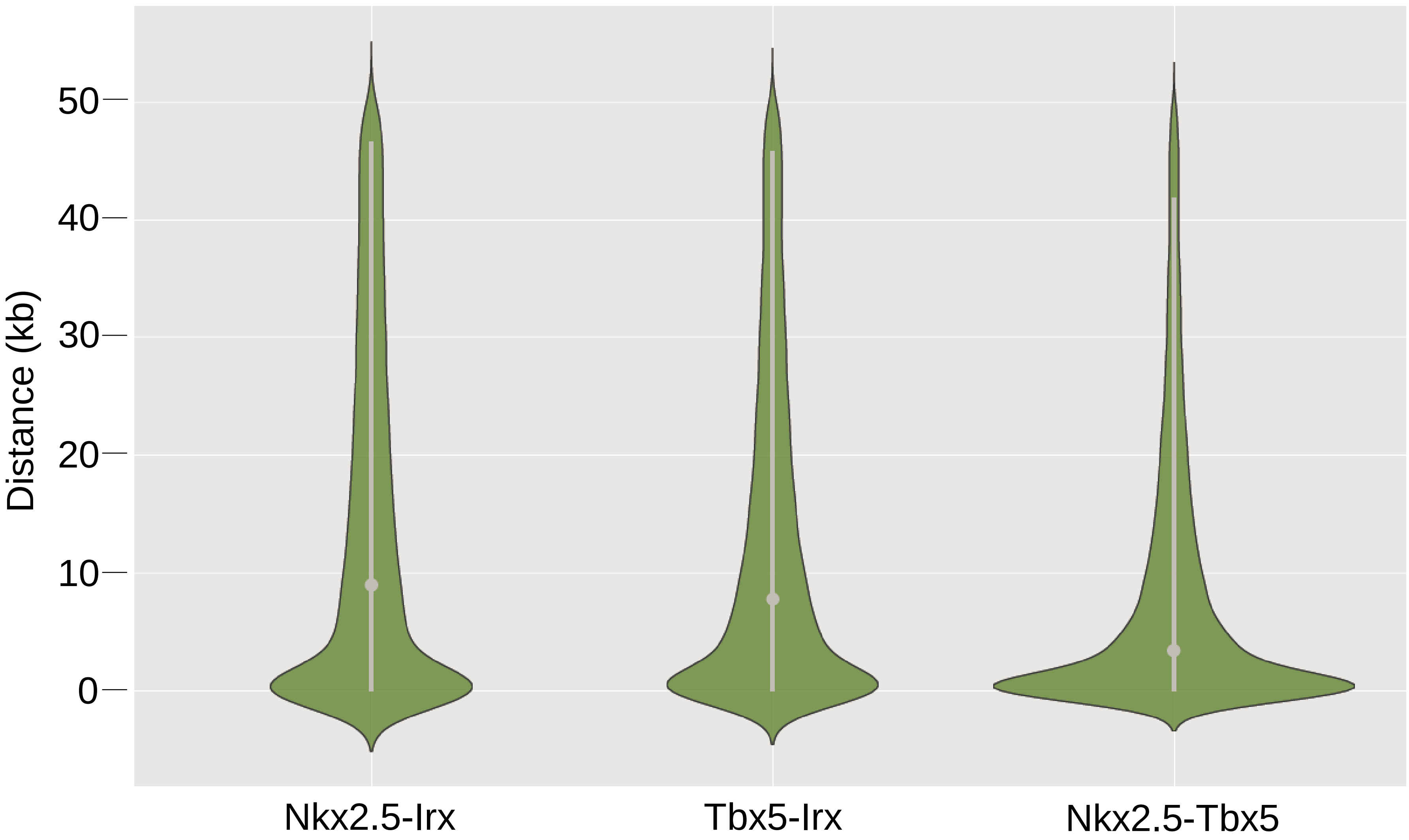

Supplementary Figure 9

a

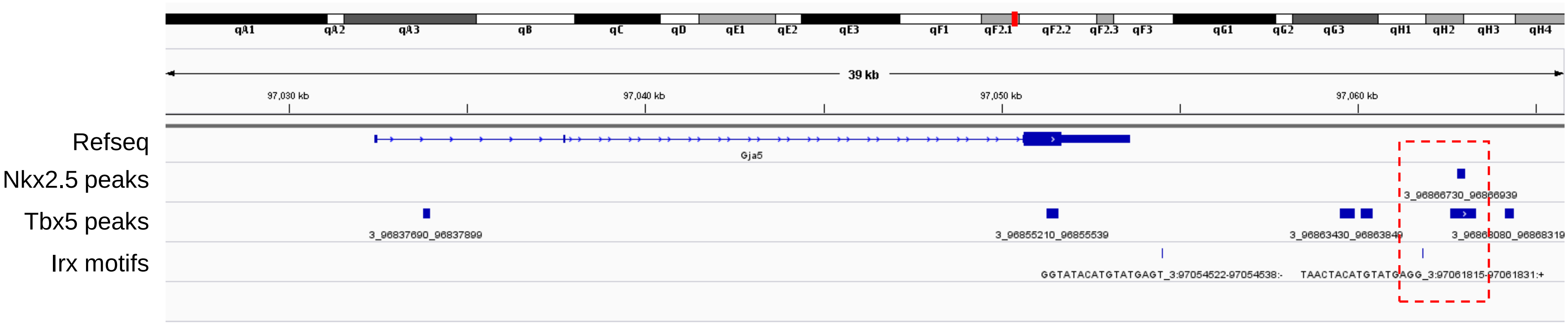

b

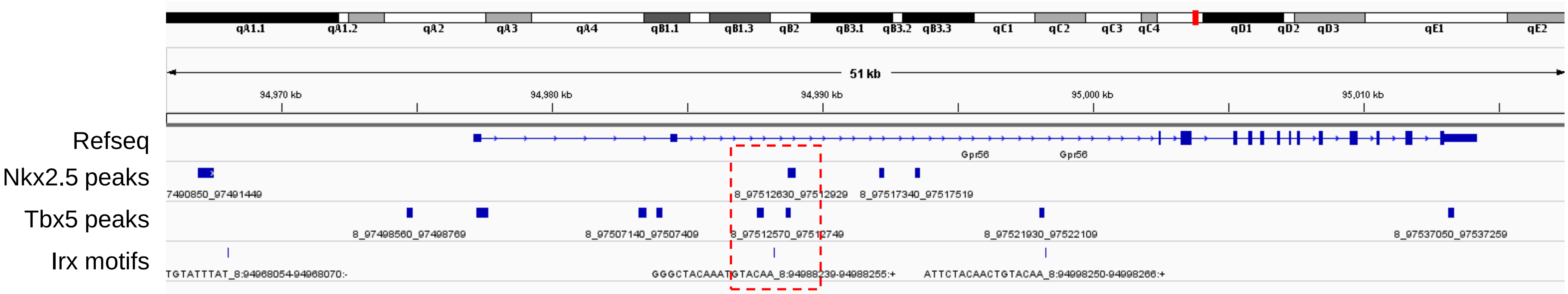

Supplement: Supplementary Information [file srep19197-s1.pdf]
